# Silver Ion Chelated Melamine–Cellulose Nanocomposite Aerogel with Highly Efficient Absorption of Oils and Organic Solvents

**DOI:** 10.3390/gels11090683

**Published:** 2025-08-27

**Authors:** Hongbo Gu, Xiwei Tan, Tao Yu, Yingqian Huang, Juan Zhang, Qixiang Zhang, Xiqiu Zhao

**Affiliations:** 1 Shanghai Key Lab of Chemical Assessment and Sustainability, School of Chemical Science and Engineering, Tongji University, Shanghai 200092, China; 2231029@tongji.edu.cn; 2 School of Aerospace Engineering and Applied Mechanics, Tongji University, 1239 Siping Road, Shanghai 200092, China; 3 Army Engineering University of PLA, Nanjing 210001, China; a19852107919@126.com (Y.H.); zhangj426601@126.com (J.Z.); zqxcumt@126.com (Q.Z.)

**Keywords:** nanocomposite aerogel, melamine, nanocellulose, oils absorption

## Abstract

As the world develops technologically and economically, the issue of environmental pollution has garnered increasing attention. Cellulose, the most abundant natural polymer on Earth, offers a promising solution. Cellulose-based aerogels are cost-effective, environmentally friendly, and effective at absorbing oil and organic pollutants. However, their absorption capacity is still limited. It requires the new method to modify the structure of cellulose aerogel and address this problem. In this work, by chelating silver ions with melamine and cellulose nanofibers through freeze-drying, the melamine–cellulose nanocomposite (Ag^+^-MNC) aerogels are prepared, which are tested for their ability to absorb various oils and organic solvents. The effects of nanocellulose and Ag^+^ concentrations on the absorption performance of nanocomposite aerogel are evaluated. The results show that the Ag^+^-MNC aerogels possess the very high absorption capacities with the values of 157.58 ± 3.38, 199.47 ± 5.65, 120.96 ± 7.04, 239.40 ± 7.41, 142.83 ± 5.30, 103.30 ± 4.73, 124.03 ± 4.05, and 118.95 ± 6.53 g/g for acetone, ethyl acetate, cyclohexane, dichloromethane, ethanol, kerosene, pump oil, and waste pump oil, respectively, which are 419%, 584%, 248%, 175%, 505%, 180%, 293%, and 268% higher than pure nanocellulose aerogels. Our Ag^+^-MNC aerogel has potential application in the absorption of oils and organic solvents.

## 1. Introduction

The rapid growth of modern industry has contributed to significant economic progress, but it has also led to increased water pollution [1]. Water pollutants primarily include oils and organic solvents, inorganic substances, nutrients, microbiological pollutants, suspended solids, radioactive materials, and plastics [2]. Among them, oils and organic solvents are the second-largest contributors to aquatic pollution [3]. Most of them are often poorly soluble in water and can form an oily layer on the surface of water body, further inhibiting oxygen exchange and impairing the respiration of aquatic organisms [4]. Normally, absorption technique is used to treat oils and organic solvents in wastewater for its simplicity [5]. Especially, as a new type of material prepared from organic, inorganic, or composite materials [6], aerogels with advantages of low density, high specific surface area, and high porosity exhibit excellent absorption properties compared to nanopowders, nanomembranes, and nanoblocks in the wastewater treatment [7,8,9]. Recently, aerogels from natural polymers and their derivatives have emerged as a promising solution for absorption of oil and organic solvent [10].

Cellulose, as a natural polymer and the main component of plant cell walls, is one of the most abundant organic compounds on Earth [11]. Owing to its low cost, high stability, and renewable properties, cellulose aerogels could be applied in antisepsis [12], catalysis [13], heat resistance [14], radiation absorption [15], pollutant adsorption [16], and bioengineering [17]. However, their mechanical strength and absorption capacity are limited, requiring modification for enhancing their overall performance. For instance, Benito-González et al. [18] enhanced the mechanical properties of nanocellulose aerogels by coating them with polylactic acid (PLA), which resulted in a 10-fold increase in compression stress compared to the unmodified aerogels. Similarly, Li et al. [19] prepared aerogels by incorporating UiO-66 into a crosslinked structure of cellulose and carboxymethyl cellulose, achieving an absorption capacity up to 210 g/g-3 times higher than that of pure nanocellulose aerogel.

Melamine is a widely used industrial raw material in the manufacture of melamine formaldehyde resins, with excellent heat and chemical resistance [20,21]. Its molecular structure contains three free amino groups (-NH_2_) and three cyano groups (-C≡N), making it suitable for metal ion adsorption [22,23]. In addition, it also could be adapted for absorption of oils or organic solvents. For example, Zhang et al. [24] utilized Zr^4+^ to alter melamine sponges, which exhibited absorption capacities ranging from 70–181 g/g for ether, acetone, cyclohexane, ethanol, toluene, methylpyrrolidone, soybean oil, methylene chloride, n-hexane, and tetrachloromethane. Patil et al. [25] applied the cucurbit [6] uril to fabricate hydrophobic melamine sponges by a simple infiltration method, which demonstrated absorption capacities ranging from 53–112 g/g for hexane, xylene, acetone, dichloromethane, diesel, petrol, edible oil, crude oil, pump oil, and chloroform.

By combining the advantages of cellulose and melamine, in the present work, we report an Ag^+^ chelated melamine–cellulose nanocomposite (Ag^+^-MNC) aerogel with excellent absorption properties for oils and organic solvents. The different weight percentages of nanocellulose solutions (0.025–0.15 wt%) and various silver nitrate solution concentrations are employed to obtain the optimal fabrication condition for Ag^+^-MNC aerogels. Their absorption capacity for acetone, ethyl acetate, cyclohexane, dichloromethane, ethanol, paraffin, pump oil, and waste pump oil are examined, which is significantly improved compared to both pure cellulose (NC) aerogels and Ag^+^ chelated melamine (Ag^+^-MA) aerogels.

## 2. Results and Discussion

### 2.1. Optimal Condition for Preparing Ag^+^-MNC Aerogel

**Figure 1:**
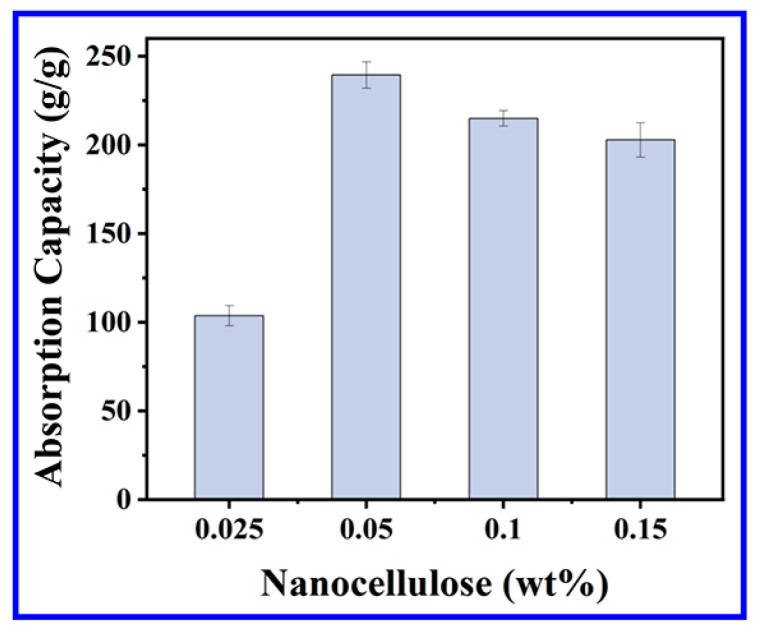
Absorption capacity of dichloromethane of Ag^+^-MNC aerogels prepared with different mass concentrations of cellulose nanofiber solution.

To confirm the optimal synthesis condition of Ag^+^-MNC aerogels, four sets of samples were synthesized by using NC solutions with weight percentages ranging from 0.025–0.15 wt%. Dichloromethane was selected as the absorbate for evaluation of their absorption capacity. Figure 1 shows the absorption capacities of Ag^+^-MNC aerogels synthesized from 0.025, 0.05, 0.10, and 0.15 wt% NC solutions to dichloromethane, which are 103.73 ± 5.76, 239.40 ± 7.41, 214.94 ± 4.37, and 202.81 ± 9.62 g/g, respectively. Therefore, 0.05 wt% of NC solution is the optimal NC concentration for the synthesis of Ag^+^-MNC aerogels. We have found that the Ag^+^-MNC aerogels prepared with different Ag^+^ concentrations exhibit the various optimal absorption capacities to different oils and organic solvents, so we have decided to discuss it in detail in the following section on absorption capacity.

### 2.2. Structure Characterizations

**Scheme 1:**
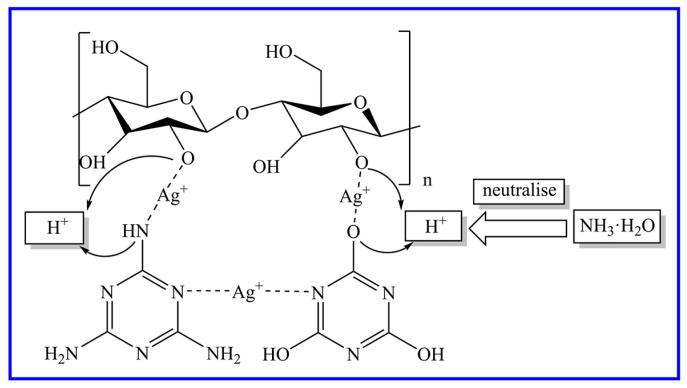
Proposed interaction between cellulose, melamine, cyanuric acid, and Ag^+^.

**Figure 2:**
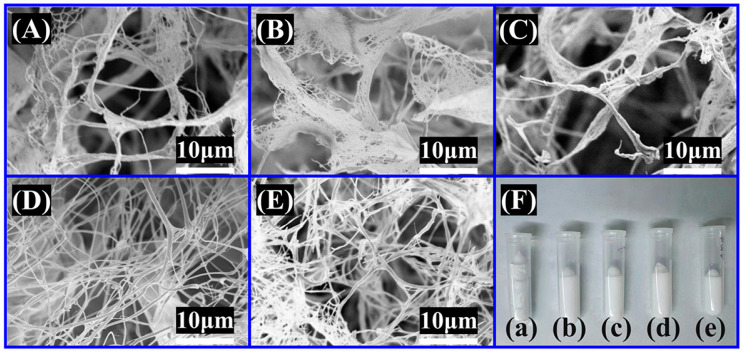
SEM images of Ag^+^-MNC aerogels with concentration of Ag^+^ of (**A**) 20 mmol/L, (**B**) 25 mmol/L, (**C**) 30 mmol/L, (**D**) NC aerogels, (**E**) Ag^+^-MA aerogels, (**F**) Digital photo of (a) NC aerogels, Ag^+^-MNC aerogels with concentration of Ag^+^ (b) 20 mmol/L, (c) 25 mmol/L, (d) 30 mmol/L, (e) Ag^+^-MA aerogels.

The structure of Ag^+^-MNC aerogel is characterized by SEM, FTIR, and XRD. The proposed formation mechanism of Ag^+^-MNC aerogel is listed in Scheme 1. Ammonia neutralizes the protons, and, subsequently, Ag^+^ is incorporated into the framework through coordination bonds with nitrogen and oxygen atoms in melamine, cyanuric acid, and nanocellulose. This interaction promotes the complexation, interconnection, and self-assembly of the material into a multilayer hydrogel and then freeze-dried to form the Ag^+^-MNC aerogel. SEM images of Ag^+^-MNC aerogels with different concentrations of Ag^+^ are given in Figure 2A–C. It is noticed that the internal pore sizes of Ag^+^-MNC aerogels are changed with the different concentrations of Ag^+^. As the concentration of Ag^+^ increases, the complexation of aerogel becomes stronger, leading to larger pores. Compared with pure NC aerogels in Figure 2D and Ag^+^-MA aerogels in Figure 2E, the Ag^+^-MNC aerogels exhibit a reticulated fibrous structure. Figure 2F reveals the digital photo of various aerogels. It is apparent that the color differences are minimal, but the NC aerogels are less smooth than Ag^+^-MNC aerogels and Ag^+^-MA aerogels.

**Figure 3:**
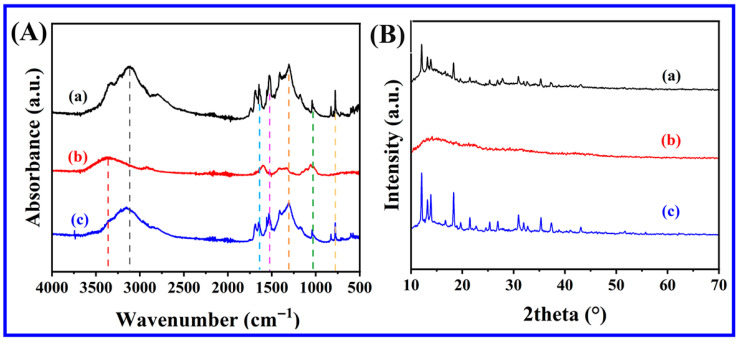
(**A**) FTIR spectra and (**B**) XRD patterns of (a) Ag^+^-MA aerogels, (b) NC aerogels, (c) Ag^+^-MNC aerogels. Dash lines in (**A**) represent corresponding characteristic peaks.

The FTIR spectra of Ag^+^-MA aerogels, NC aerogels, and Ag^+^-MNC aerogels are shown in Figure 3A. In the FTIR spectrum of Ag^+^-MA aerogels, Figure 3A (a), the peaks at 3118, 1640, and 1521 cm^−1^ are ascribed to the stretching vibration of conjugated -NH_2_, triazine ring, -C=N, and the peak at 780 cm^−1^ is the out-of-plane bending vibration of -NH_2_. In the FTIR spectrum of pure NC aerogels, Figure 3A (b), the peaks at 1033 and 3360 cm^−1^ correspond to C-O stretching vibration and hydroxyl stretching vibration generated from hydrogen bonding. These characteristic peaks located at 3360, 3318, 1640, 1521, 1033, and 780 cm^−1^ all appear in the FTIR spectrum of Ag^+^-MNC aerogel (Figure 3A (c)), indicating that both melamine and nanocellulose are present in Ag^+^-MNC aerogel.

The XRD patterns of Ag^+^-MNC aerogels, NC aerogels, and Ag^+^-MA aerogels are presented in Figure 3B. In the XRD pattern of Ag^+^-MA aerogels, Figure 3B (a), the most pronounced peaks are located at 11.6, 13.2, 14.5, and 18.0°, which are assigned to the (110), (200), (220), and (202) crystallographic planes. In the XRD pattern of NC aerogels, Figure 3B (b), the peaks located at 15.1 and 22.5° conform to the (110) and (200) crystallographic planes. These are the characteristic peaks in the cellulose crystalline region. The pronounced diffraction peaks of Ag^+^-MNC aerogels are located at 11.6, 13.2, 14.5, 15.1, and 18.0° [26], Figure 3B (c), which are the characteristic peaks from both cellulose and Ag^+^-MA. These results also suggest that both melamine and nanocellulose existed in the Ag^+^-MNC aerogels.

The BET analysis of pure NC aerogel, Ag^+^-MA aerogel, and Ag^+^-MNC aerogels with Ag^+^ concentrations of 20, 25, and 30 mmol/L, respectively, was carried out. As shown in Table S3, the resulting BET specific surface area of pure NC aerogels, Ag^+^-MA aerogels, and Ag^+^-MNC aerogels with Ag^+^ concentration of 20, 25, and 30 mmol/L is 21.21, 11.09, 43.62, 32.65, and 23.46 m^2^∙g^−1^, respectively. As the Ag^+^ concentration increases, the BET specific surface area of Ag^+^-MNC aerogels is gradually decreased. However, they are still higher than that of pure NC aerogels, Ag^+^-MA aerogels. The porosity of pure NC aerogels, Ag^+^-MA aerogels, and Ag^+^-MNC aerogels with Ag^+^ concentration of 20, 25, and 30 mmol/L is 98.54, 96.27, 99.25, 99.13, and 99.01%, respectively, in which the Ag^+^-MNC aerogels possess the highest porosity, surpassing both pure NC aerogels, Ag^+^-MA aerogels.

### 2.3. Thermal Stability

**Figure 4:**
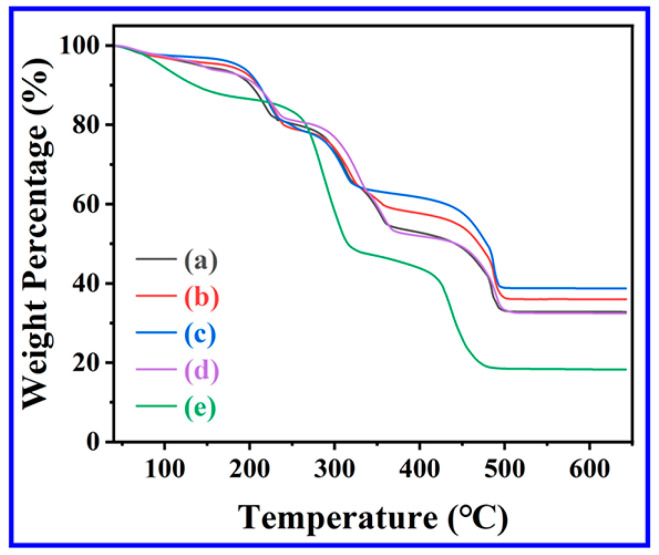
TGA curves of Ag^+^-MNC aerogel with Ag^+^ concentration of (a) concentration of Ag^+^ is 20 mmol/L, (b) 25 mmol/L, (c) 30 mmol/L, (d) Ag^+^-MA aerogel, (e) NC aerogel.

Melamine-based materials usually have better thermal stability, which gives a competitive advantage in heat resistance [27,28]. Figure 4 displays the TGA curves of NC aerogels, Ag^+^-MA aerogels, and Ag^+^-MNC aerogels with different concentrations of Ag^+^. In the TGA curve of NC aerogel, Figure 4 (e), the initial slight weight loss of NC aerogel below 100 °C is due to water evaporation. The first major weight loss from 250 to 320 °C is mainly due to the decomposition of their net structure. The second significant weight loss from 320 to 480 °C is caused by the thermal decomposition of the carbon skeleton [29]. Except for NC aerogels, the Ag^+^-MA aerogels and Ag^+^-MNC aerogels disclose the similar decomposition profiles with three apparent weight loss stages. Obviously, the thermal stability of Ag^+^-MA aerogels and Ag^+^-MNC aerogels is better than that of NC aerogels by evaluating the initial decomposition temperature, implying that the overall thermal stability of Ag^+^-MA aerogels and Ag^+^-MNC aerogels is improved by the addition of melamine and the complexation of Ag^+^. For Ag^+^-MNC aerogels, Figure 4 (a)–(c), the initial weight loss in the first stage from 190 to 230 °C is due to the disruption of the Ag^+^ complexation structure. The weight loss in the second stage that starts from ~230 °C is mainly due to the breakdown of functional groups and the release of NH_3_ (g) and HCN (g) [30]. The final weight loss in the third stage from ~360 to 490 °C corresponds to the destruction of the triazine ring structure and carbon skeleton from the cellulose. The final weight residue in the Ag^+^-MNC aerogels varies with the concentrations of Ag^+^. The thermal decomposition behavior of Ag^+^-MA aerogels is not apparently different from that of Ag^+^-MNC aerogels with the same concentration of Ag^+^, Figure 4 (d). Its TGA curve is only slightly different from those of Ag^+^-MNC aerogels in the second stage, which is supposed to be due to the absence of nanocellulose, resulting in a slight increase in the thermal stability compared to that of Ag^+^-MNC aerogels.

### 2.4. Absorption Properties

**Figure 5:**
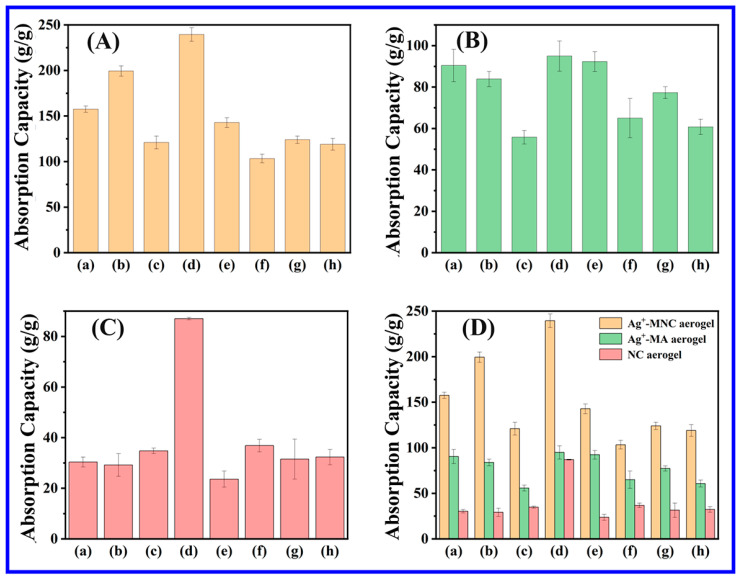
(**A**) Absorption capacities of Ag^+^-MNC aerogel, (**B**) Absorption capacities of Ag^+^-MA aerogel, (**C**) Absorption capacities of NC aerogel, and (**D**) Comparison of absorption capacities of Ag^+^-MNC aerogel, Ag^+^-MA aerogel and NC aerogel to (a) acetone, (b) ethyl acetate, (c) cyclohexane, (d) dichloromethane, (e) ethanol, (f) kerosene, (g) pump oil, (h) waste pump oil.

Acetone, ethyl acetate, cyclohexane, dichloromethane, ethanol, kerosene, pump oil, and waste pump oil were picked up to test the absorption performance of Ag^+^-MNC aerogels. For comparison, the absorption capacities of Ag^+^-MA aerogel and NC aerogel on the above organic solvents and oils were also tested. The specific absorption capacities of these aerogels are shown in Figure 5 and Table 1. Figure 5A displays the optimal absorption capacities of Ag^+^-MNC aerogel for acetone, ethyl acetate, cyclohexane, dichloromethane, ethanol, kerosene, pump oil, and waste pump oil are 157.58 ± 3.38, 199.47 ± 5.65, 120.96 ± 7.04, 239.40 ± 7.41, 142.83 ± 5.30, 103.30 ± 4.73, 124.03 ± 4.05, and 118.95 ± 6.53 g/g, separately. The absorption capacities are varied for Ag^+^-MNC aerogel with different concentrations of Ag^+^. As shown in Table S1, Ag^+^-MNC aerogel prepared with 20 mmol/L Ag^+^ expresses the best absorption capacity to ethyl acetate, cyclohexane, pump oil, and waste pump oil. Ag^+^-MNC aerogel prepared with 25 mmol/L Ag^+^ evidences the best absorption capacity to acetone, ethanol, and kerosene. Ag^+^-MNC aerogel prepared with 30 mmol/L Ag^+^ manifests the best absorption capacity to dichloromethane. Figure 5B discloses the absorption capacities of Ag^+^-MA aerogel to acetone, ethyl acetate, cyclohexane, dichloromethane, ethanol, kerosene, pump oil, and waste pump oil are 90.39 ± 7.79, 83.87 ± 3.71, 55.79 ± 3.27, 94.93 ± 7.32, 92.30 ± 4.80, 64.99 ± 9.46, 77.27 ± 2.83, and 60.76 ± 3.71 g/g, accordingly. Figure 5C reveals the absorption capacities of NC aerogel to acetone, ethyl acetate, cyclohexane, dichloromethane, ethanol, kerosene, pump oil, and waste pump oil are 30.36 ± 1.95, 29.17 ± 4.49, 34.78 ± 1.06, 87.03 ± 0.46, 23.60 ± 3.20, 36.85 ± 2.46, 31.52 ± 7.89, and 32.31 ± 3.05 g/g, respectively. Figure 5D provides the comparable absorption capacity of these three aerogels to series of oils and organic solvents. It could be concluded that the absorption capacities of Ag^+^-MNC aerogel for acetone, ethyl acetate, cyclohexane, dichloromethane, ethanol, kerosene, pump oil, and waste pump oil are much higher than Ag^+^-MA aerogel and NC aerogel, which are 74, 138, 117, 152, 55, 59, 61, and 96% higher than Ag^+^-MA aerogel, and 419, 584, 248, 175, 505, 180, 293, and 268% higher than NC aerogel, accordingly. This connotes that the addition of melamine and chelation with Ag^+^ could significantly improve the absorption capacity of NC aerogel. A comparison of the absorption performance of various oil-adsorbing materials in the literature is presented in Table 2. It can be summarized that our Ag^+^-MNC aerogels have relatively excellent absorption properties for oils and organic solvents than other reported materials. The absorption process is attributed to the pore structure of aerogel since the main role for the absorption of oils and organic solvents by aerogel is from the capillary siphon effect and the addition of melamine and chelation with Ag^+^ significant alter the pore structure of aerogel, which rapidly reaches absorption equilibrium in a very short period of time by the effect of siphoning, so the absorption kinetic is not discussed here.

In order to verify that there is no interaction between the hydroxyl groups in ethyl acetate and Ag^+^-MNC aerogel, the weight difference before and after absorption was performed. As shown in Figure S2A, the Ag^+^-MNC aerogel is weighed as 0.00847 g before absorption of ethyl acetate. Then, after absorption of ethyl acetate and drying, this aerogel is weighed again as 0.00835 g in Figure S2B. These results show that the weight has no significant change after absorption of ethyl acetate, which confirms that there is no interaction between ethyl acetate and Ag^+^-MNC aerogel. The structural formula of the remaining organic solvents is given in Table S2. Their structural formula indicates that there is no interaction between them and Ag^+^-MNC aerogel. Kerosene and pump oil are hydrocarbons and also have no interaction with Ag^+^-MNC aerogel. The varying absorption capacities of these aerogels for different oils and organic solvents may primarily be attributed to the differences in their viscosity and volatility (see Table S2).

## 3. Conclusions

In this work, we have prepared Ag^+^-MNC aerogels with different pore structures compared to pure NC aerogels and Ag^+^-MA aerogels. The highest absorption capacity of the aerogel is achieved at a concentration of 0.05 wt% of NC solution. The absorption capacity of Ag^+^-MNC aerogels for oils and organic solvents evinces a substantial increase relative to NC aerogels and Ag^+^-MA aerogels, with their values to acetone, ethyl acetate, cyclohexane, dichloromethane, ethanol, kerosene, pump oil, and waste pump oil of 157.58 ± 3.38, 199.47 ± 5.65, 120.96 ± 7.04, 239.40 ± 7.41, 142.83 ± 5.30, 103.30 ± 4.73, 124.03 ± 4.05, and 118.95 ± 6.53 g/g, respectively. The results highlight the superior performance of Ag^+^-MNC aerogels in the absorption of oils and organic solvents, which gives them potential for application in environmental remediation. The higher BET specific surface area and porosity make contributions to the higher absorption property of Ag^+^-MNC aerogels relative to pure NC aerogels and Ag^+^-MA aerogels. Even though our as-prepared Ag^+^-MNC aerogels are biodegradable and biocompatible, the relatively low mechanical strength limits its regenerations and reusability. The future work will consider the method to strengthen the mechanical property of these kinds of aerogels to make them more efficiently be utilized in the real-world applicability.

## 4. Materials and Methods

### 4.1. Materials

Cellulose nanofiber (NC, 99.6%, derived from cotton) with the diameter of 4–10 nm and length of 1–3 μm was obtained from Guilin Qihong Technology Co., Ltd. (Guilin, China). Melamine (99%) and Cyanuric acid (98%) were offered by Adamas Reagents Co., Ltd. (Shanghai, China) Ammonia (25–28 wt%), acetone (≥99.5%), and silver nitrate (≥99.8%) were gained from Shanghai Hushi Laboratorial Equipment Co., Ltd. (Shanghai, China). Pump oil was gained from Edwards Technologies Trading Co., Ltd. (Shanghai, China). Anhydrous ethanol (≥99.7%) was provided by Shanghai Kuling Superfine Chemical Industry Co., Ltd. (Shanghai, China). Kerosene (composed of 28–48% alkanes, 20–50% or 8–15% aromatics, 1–6% unsaturated hydrocarbons, 17–44% cyclic hydrocarbons) was acquired from Alfa Aesar (China) Chemical Co., Ltd. (Shanghai, China). Dichloromethane (99.5%), ethyl acetate (99%), and cyclohexane (99.7%) were attained from Shanghai Macklin Biochemical Co., Ltd. (Shanghai, China). Unless otherwise specified, all the chemicals were used as-received.

### 4.2. Preparation of Ag^+^-MNC Aerogel

The synthesis steps of Ag^+^-MNC aerogel are as follows. Firstly, the 0.05–0.15 wt% of nanocellulose solution, 20 mmol/L melamine solution, 20 mmol/L cyanuric acid solution, 10 wt% ammonia solution, and 20 mmol/L to 30 mmol/L silver nitrate solution were prepared accordingly. Then, 0.5 mL of melamine solution, 0.5 mL of cyanuric acid solution, and 0.2 mL of ammonia solution were added to 0.5 mL of nanocellulose solution in sequence at room temperature to form the mixed solution. After ultrasonication for 10 s, 1.0 mL of silver nitrate solution was introduced to the above mixed solution to obtain the hydrogel. Finally, this hydrogel was immersed in the liquid nitrogen for 10 min and freeze-dried for 24 h to acquire the Ag^+^-MNC aerogel. NC aerogel was also synthesized for comparison. Ag^+^-MA aerogel was also synthesized by the same method without the addition of NC solution.

### 4.3. Characterizations

The microstructure of aerogels was examined on a scanning electron microscope (SEM, JSM-7900F system, JEOL, Tokyo, Japan). The chemical structure of aerogels was analyzed by Fourier Transform Infrared (FTIR) spectroscopy using a Thermo Nicolet NEXUS Infrared Spectrometer (Thermo Scientific, Waltham, USA) with an attenuated total reflection (ATR) accessory in the wavelength range of 500 to 4000 cm^−1^. The thermal stability of aerogels was performed by the thermogravimetric analysis (TGA, TGA 55, TA Instruments, Waters Corporation, Milford, MA, USA) from 30 to 650 °C with a heating rate of 20 °C min^−1^ in an air environment. Contact angle measurement was carried out on a JC2000D2G high-speed contact angle measuring instrument (Shanghai Zhongchen Digital Technology Equipment Co., Ltd., Shanghai, China). The crystalline structure of aerogels was conducted on an X-ray diffractometer (XRD, D8 Advance, Bruker Corporation, Billerica, USA). The specific surface area of aerogels was determined by using the Specific Surface Area Analyzer (Tristar3020, McMurray Tick Instruments Co., Ltd., Shanghai, China).

### 4.4. Absorption Tests for Oil and Organic Solvents

The absorption capacity was tested with following steps. First, the total mass of the sealed bottle containing oil or organic solvent was weighed and noted as *m*_1_. Then, a known quantity of the aerogel was added into the oil or organic solvent in the above sealed bottle by the tweezers for 5 min. Next, the absorbed aerogel was quickly removed by the tweezers to avoid evaporation of the liquid. Finally, the sealed bottle was weighed and recorded as *m*_2_; the absorption capacity *q* (g/g) was determined according to Equation (1):

(1) $$q=\frac{m_{1}-m_{2}}{m_{aerogel}}$$

where *m_aerogel_* was the weight of the aerogel.

Acetone, ethyl acetate, cyclohexane, dichloromethane, ethanol, kerosene, pump oil, and waste pump oil were chosen to test the absorption capacity of Ag^+^-MNC aerogel, NC aerogel, and Ag^+^-MA aerogel, respectively. The reported values were the average of three measurements with error bars.

## Figures and Tables

**Table 1:** Absorption capacity of Ag^+^-MNC aerogel with NC aerogel and Ag^+^-MA aerogel.

Organic Solvents and Oil	Absorption Capacities/(g/g)		
	Ag^+^-MNC Aerogel	NC Aerogel	Ag^+^-MA Aerogel
Acetone	157.58 ± 3.38	30.36 ± 1.95	90.39 ± 7.79
Ethyl acetate	199.47 ± 5.65	29.17 ± 4.49	83.87 ± 3.71
Cyclohexane	120.96 ± 7.04	34.78 ± 1.06	55.79 ± 3.27
Dichloromethane	239.40 ± 7.41	87.03 ± 0.46	94.93 ± 7.32
Ethanol	142.83 ± 5.30	23.60 ± 3.20	92.30 ± 4.80
Kerosene	103.30 ± 4.73	36.85 ± 2.46	64.99 ± 9.46
Pump oil	124.03 ± 4.05	31.52 ± 7.89	77.27 ± 2.83
Waste pump oil	118.95 ± 6.53	32.31 ± 3.05	60.76 ± 3.71

**Table 2:** Comparison of Ag^+^-MNC aerogel with other advanced materials for absorption capacity of oil and organic solvents.

Materials	Absorption Capacities/(g/g)
Three-dimensional graphene-based aerogel [31]	Pump oil: 191; Dichloromethane: 179 Acetone: 114; Cyclohexane: 110; Kerosene: 100
Bacterial cellulose biomass aerogel [32]	Pump oil: 100; Acetone: 66
PVA/CNF/MTMS aerogel [33]	Acetone: 56; Ethanol: 52; Pump oil: 71; Kerosene: 55
PVA-based PNP/CNTs aerogel [34]	Ethanol: 35
Carrageenan cross-linked graphene sponge [35]	Acetone: 26; Dichloromethane: 28
DCMC cross-linked collagen aerogel [36]	Kerosene: 22; Ethyl acetate: 28
Sponge-like chitosan/CNTs/silica composite [37]	Cyclohexane: 9; Kerosene: 10
PANF/CNT aerogel [38]	Kerosene: 40; Dichloromethane: 53
Bio-inspired castor oil modified cellulose aerogel [39]	Dichloromethane: 114
Polybenzoxazine/reduced graphene oxide wrapped-cellulose sponge [40]	Ethanol: 72; Acetone: 95; Ethyl acetate: 100
Superhydrophobic melamine sponge [41]	Dichloromethane: 135; Ethyl acetate: 107
NCA/OA/Fe_3_O_4_ aerogel [42]	Cyclohexane: 68; Ethyl acetate: 56; Pump oil: 33
NC/Al_2_O_3_ aerogel [43]	Dichloromethane: 118; Acetone: 85; Cyclohexane: 71; Ethyl acetate: 94; Ethanol: 90
Ag^+^-MNC aerogel (this work)	Acetone: 158; Ethyl acetate: 199; Cyclohexane: 121; Dichloromethane: 239; Ethanol: 143; Kerosene: 103; Pump oil: 124; Waste pump oil: 119

## Data Availability

All the data are available at the request of the authors. Supporting Information is in the online version.

## Supplementary Materials

The following supporting information can be downloaded at: https://www.mdpi.com/article/10.3390/gels11090683/s1, Figure S1: Contact angle measurement between Ag^+^-MNC aerogel and pump oil; Figure S2: (A) Mass of the aerogel before absorption of ethyl acetate; (B) Mass of the aerogel after absorption and drying of ethyl acetate; Table S1: Absorption capacities of Ag^+^-MNC aerogels prepared using different concentrations of silver nitrate solutions for organic solvents and oils; Table S2: Viscosity of organic solvents and oils (20 °C); Table S3: BET results and porosity of different aerogels.
