# Supplementary material for: Silver Ion Chelated Melamine–Cellulose Nanocomposite Aerogel with Highly Efficient Absorption of Oils and Organic Solvents
Source: Gels. 2025 Aug 27;11(9):683. doi: 10.3390/gels11090683 (PMC12469491; doi:10.3390/gels11090683)
Supplement: Supplementary file 1 [file gels-11-00683-s001.zip › gels-3807890-supplementary.pdf]

**Supporting Information for**

# Melamine-cellulose nanocomposite aerogel with highly efficient adsorption of oils and organic solvents

Hongbo Gu,[a,]* Xiwei Tan,[a] Tao Yu,[b,*] Yingqian Huang,[c] Juan Zhang,[c] Qixiang Zhang,[c] Xiqiu Zhao[c,*]

[a]Shanghai Key Lab of Chemical Assessment and Sustainability, School of Chemical Science and Engineering, Tongji University, Shanghai 200092, P. R. China

[b]School of Aerospace Engineering and Applied Mechanics, Tongji University, 1239 Siping Road, Shanghai, 200092, P.R. China

*Corresponding author

E-mail: hongbogu2014@tongji.edu.cn
yutao@tongji.edu.cn
tianmang1981@126.com

***Preparation of $Ag^+$-MNC aerogel***

To identify the optimal synthesis conditions, $Ag^+$-MNC aerogel was synthesized with different nanocellulose and $Ag^+$ concentrations. First, 0.025-0.15 wt% NC solution, 20 mmol $L^{-1}$ of melamine solution, 20 mmol $L^{-1}$ of cyanuric acid solution, 10 wt% of ammonia solution, and 20 – 30 mmol $L^{-1}$ of silver nitrate solution were prepared, respectively. Next, 0.5 mL of melamine solution, 0.5 mL of cyanuric acid solution, and 0.2 mL of ammonia solution were sequentially added to 0.5 mL of above NC solution at room temperature. Then, the mixed solution was ultrasonic for 10 s, and 1.0 mL of silver nitrate solution was added above mixed solution to obtain a hydrogel. Finally, the hydrogel was frozen for 10 min in liquid nitrogen and then freeze-dried for 24 h to form the $Ag^+$-MNC aerogel. NC aerogel was also synthesized for comparison. The $Ag^+$-MA aerogel was fabricated as well with the same procedure without the addition of NC solution and the $Ag^+$ concentration of 20 mmol $L^{-1}$. The prepared aerogel has no compression properties, presumably due to its random porous structure.

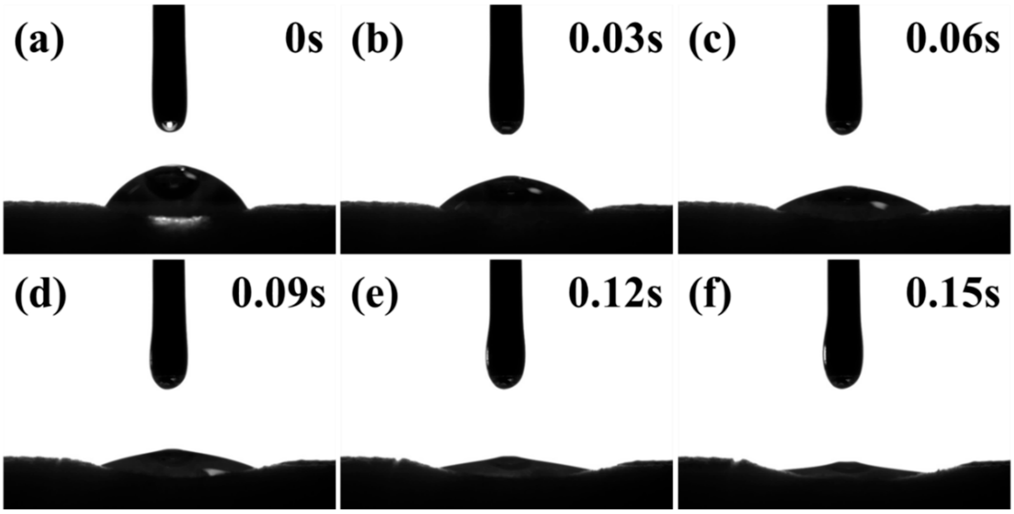


**Fig. S1** Contact angle measurement between $Ag^+$-MNC aerogel and pump oil.

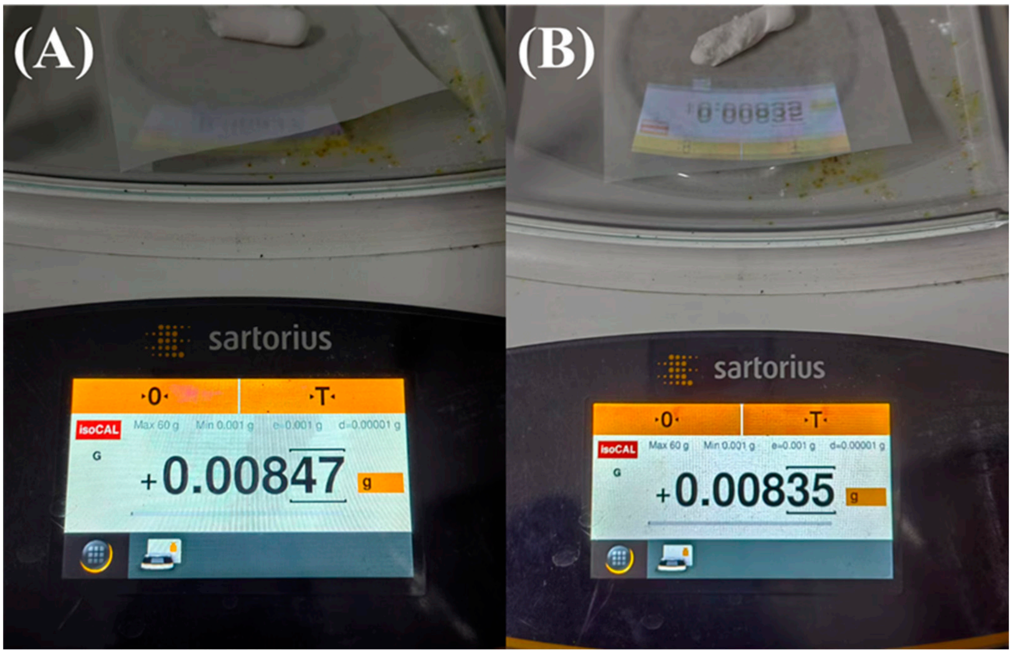


**Fig. S2** (A) Mass of the aerogel before absorption of ethyl acetate; (B) Mass of the aerogel after absorption and drying of ethyl acetate

**Table S1** Absorption capacities of $Ag^{+}$-MNC aerogels prepared using different concentrations of silver nitrate solutions for organic solvents and oils.

| **Organic solvents and oils** | **Concentration of $AgNO_3$ (aq)** | | |
|---|---|---|---|
| | **20 mmol $L^{-1}$** | **25 mmol $L^{-1}$** | **30 mmol $L^{-1}$** |
| Acetone | 155.21±2.35 | 157.58±3.38 | 105.13±3.04 |
| Ethyl acetate | 199.47±5.65 | 115.31±5.01 | 100.89±7.89 |
| Cyclohexane | 120.96±7.04 | 83.53±8.19 | 60.94±9.11 |
| Dichloromethane | 186.20±8.88 | 167.25±8.03 | 239.40±7.41 |
| Ethanol | 131.94±4.33 | 142.83±5.30 | 86.28±6.65 |
| Kerosene | 88.42±4.03 | 103.30±4.73 | 75.57±5.36 |
| Pump oil | 124.03±4.05 | 71.77±5.51 | 41.64±6.16 |
| Waste pump oil | 118.95±6.53 | 78.61±5.61 | 80.18±7.71 |

**Table S2** Viscosity of organic solvents and oils (20°C).

| Organic solvents and oils | Viscosity/(mPa s) | Structural formula |
|---|---|---|
| Acetone | 0.32 | 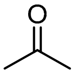 |
| Ethyl acetate | 0.45 | 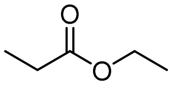 |
| Cyclohexane | 0.89 | 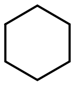 |
| Dichloromethane | 0.43 | 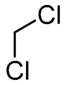 |
| Ethanol | 0.88 | 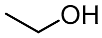 |
| Kerosene | 0.80 | --- |
| Pump oil | 0.68 | --- |

**Table S3** BET results and porosity of different aerogels.

| Aerogels | BET Specific surface area ($m^2$ $g^{-1}$) | Porosity (%) |
|---|---|---|
| $Ag^+$-MA aerogel | 21.21 | 98.54 |
| NC aerogel | 11.09 | 96.27 |
| Ag+-MNC aerogel (20 mmol/L $Ag^+$) | 43.62 | 99.25 |
| Ag+-MNC aerogel (25 mmol/L $Ag^+$) | 32.65 | 99.13 |
| Ag+-MNC aerogel (30 mmol/L $Ag^+$) | 23.46 | 99.01 |
